# Electronic Noses for Composites Surface Contamination Detection in Aerospace Industry

**DOI:** 10.3390/s17040754

**Published:** 2017-04-02

**Authors:** Saverio De Vito, Maria Lucia Miglietta, Ettore Massera, Grazia Fattoruso, Fabrizio Formisano, Tiziana Polichetti, Maria Salvato, Brigida Alfano, Elena Esposito, Girolamo Di Francia

**Affiliations:** ENEA—Italian National Agency for New Technologies, Energy and Sustainable Economic Development, PV and Smart Network Division, C.R. ENEA Portici, P.le E. Fermi, 1, 80055 Portici, Italy; mara.miglietta@enea.it (M.L.M.); ettore.massera@enea.it (E.M.); grazia.fattoruso@enea.it (G.F.); fabrizio.formisano@enea.it (F.F.); tiziana.polichetti@enea.it (T.P.); maria.salvato@enea.it (M.S.); brigida.alfano@enea.it (B.A.); elena.esposito@enea.it (E.E.)

**Keywords:** NDTs, composites, surface contamination detection, electronic noses, aerospace industry, pattern recognition

## Abstract

The full exploitation of Composite Fiber Reinforced Polymers (CFRP) in so-called *green aircrafts* design is still limited by the lack of adequate quality assurance procedures for checking the adhesive bonding assembly, especially in load-critical primary structures. In this respect, contamination of the CFRP panel surface is of significant concern since it may severely affect the bonding and the mechanical properties of the joint. During the last years, the authors have developed and tested an electronic nose as a non-destructive tool for pre-bonding surface inspection for contaminants detection, identification and quantification. Several sensors and sampling architectures have been screened in view of the high Technology Readiness Level (TRL) scenarios requirements. Ad-hoc pattern recognition systems have also been devised to ensure a fast and reliable assessment of the contamination status, by combining real time classifiers and the implementation of a suitable rejection option. Results show that e-noses could be used as first line low cost Non Destructive Test (NDT) tool in aerospace CFRP assembly and maintenance scenarios.

## 1. Introduction

Low cost analysis of complex gas phase chemical mixtures has been made possible by the so-called electronic nose architecture that has already found application in several fields whenever mixture detection, identification and/or quantification is concerned, including medical diagnostic, environmental monitoring, food industry, etc. [1]. However, application to even wider industrial scenarios seems at present mainly limited by stability and repeatability issues [2]. Indeed, sensors fabrication process outcome is still subject to a significant variability that hampers the use of a shared calibration function, thus requiring an ad hoc calibration procedure for each electronic nose or the development of calibration transfer strategies [3,4]. Furthermore, drift effects related to ageing and poisoning of the sensors as well as the influence of environmental parameters (mostly Temperature and Relative Humidity abbreviated respectively with T and RH) have prevented their wide use in industrial high valued production fields for failing operative requirements. Still, most of the e-nose applicative research is targeted to liquid/solid samples headspace analysis in controlled environment, actually underestimating the challenges of on-field applicative scenarios. However, the e-nose characteristics and, specifically, the low overall costs (buying, operation and maintenance) make it a very appealing choice for developing industrial grade Non Destructive Test (NDT) tools [5]. The specific requirements for this application field include high reliability, fast response, low cost and the possibility to be operated by unskilled workforce. Some of them, including the need for operation in uncontrolled or even harsh environments, however, may prove very challenging for the above-mentioned issues.

Conversely, the need for NDT technologies that can cope with new challenges of the so-called lightweight aircraft industry is steadily growing along with the requests for novel integrated health monitoring systems [6,7]. Traditionally, transport industry and particularly the Aerospace industry have strongly relied on NDT tools to guarantee the safety of operations and the efficiency of the maintenance activities. Nowadays, industries are challenged by the revolution of ubiquitous adoption of composites for aircraft parts since composite can guarantee a strong reduction in the overall aircraft weight, thus reducing energy consumption and boosting efficiency. The adoption of light-weight composite materials (CFRP—Carbon Fiber Reinforced Polymers) for primary structural components, is a major milestone and may account for a significant reduction of per-mile-passenger transport costs deriving from a dramatic increase in cost efficiency for ground operations (up to 50%), a reduction in fuel usage (up to 20%) and, consequently, a CO_2_ emission rate reduction at fleet level of up to 15% [8,9]. A significant fraction of these savings comes from the assembly procedure of CFRP panels that requires, however, a completely new assembly and maintenance concept. Composite parts are not assembled in fact by riveting but instead, by adhesive bonding, a critical process that requires an almost perfect cleanliness state of the surfaces to bond, in order to avoid affecting the mechanical properties of the assembly itself [10]. Contamination may occur due to different processes that the CFRP panel may undergo during assembly or operative life. Examples vary from the hydraulic oils used for power transmission to the de-icing fluids employed on runways for safety purposes particularly in icy climate, causing in both cases chemical damages, as well as release agents utilized during CFRP molding, producing a physical screening that jeopardizes the distribution of the adhesive on the composite panel surface [11]. Thermal degradation of the surface may also affect the adhesion properties of the panel, consequently compromising the mechanical strength of the bond.

The lack of a verified procedure for assessing the quality of an adhesively bonded aircraft joint is slowing down the wider adoption of CFRP for primary structures [12]. These parts are of paramount relevance for the structural integrity of the aircraft and so their bonding should be ensured at the highest mechanical quality. For these reasons, aircraft industry stakeholders are focusing on developing a suitable NDT tool for guaranteeing the quality of adhesive bonds. European Commission actively contributes to these efforts, by means of its Research Framework Programmes and the CleanSky Joint Technical Initiative. Actually, this group and its partners benefitted from EU funding for the development of an electronic nose solution for adhesive bonding quality assessment under the projects named ICARO (JTI CLean Sky, [13]), ENCOMB (EU FP7, [14]) and COMBONDT (H2020, [9]). During these projects, the amount of performance degradation due to surface contamination has been investigated. Markatos et al. have measured the mechanical parameters of contaminated CFRP panels adhesive bonds [10,11,12] showing a reduction in the fracture toughness (GIC) in excess of 25% for Skydrol^®^500-B contamination and of more than 60% for relevant release agent contamination. Residual contamination levels contribute significantly in predicting the mechanical strength of the bond. Markatos et al., for instance, have shown that 7% Si remaining on the surface after a contamination event with release agents can be responsible for a total lack of adhesion [11].

Several technologies have been screened for their potential capability to assess the bond quality via NDT tools [15,16,17,18]. Some of them, including the electronic nose technology provided by the authors’ group, have been selected as suitable tools for quality assessment tests and are now investigated for their use at a higher Technology Readiness Level (TRL). Of course, the faced scenario resulted very challenging for the electronic nose technology and required the design of ad-hoc sampling and measurement subsystems as well as the careful selection of an ad-hoc sensor array and the design of proper machine learning algorithms in order to cope with the fast and accurate assessment needs of this safety critical application field. Several sensors have been tested by using commercially available e-nose as well as designing an ad-hoc open source platform capable to host simultaneously devices based on different technologies [5] and IR emitters have been employed for increasing the surface desorption. On line confidence based multiclassifier systems have been designed and tested to provide real time readings along with confidence ratings in order to detect, identify and quantify the contamination level [5,19]. This paper collects the most significant results obtained during this development process and presents the most recent findings obtained by the last e-nose version in the new challenging high TRL testing scenarios as defined by the COMBONDT project. In particular, in Section 2, the architecture of the involved electronic noses is outlined; in Section 3, the experimental contamination and measurement procedure is outlined for both Low and High TRL scenarios; and Section 4 describes the structure of the developed pattern recognition systems and the obtained results.

## 2. Electronic Noses Platforms

### 2.1. The GDA2 Electronic Nose

**Figure 1:**
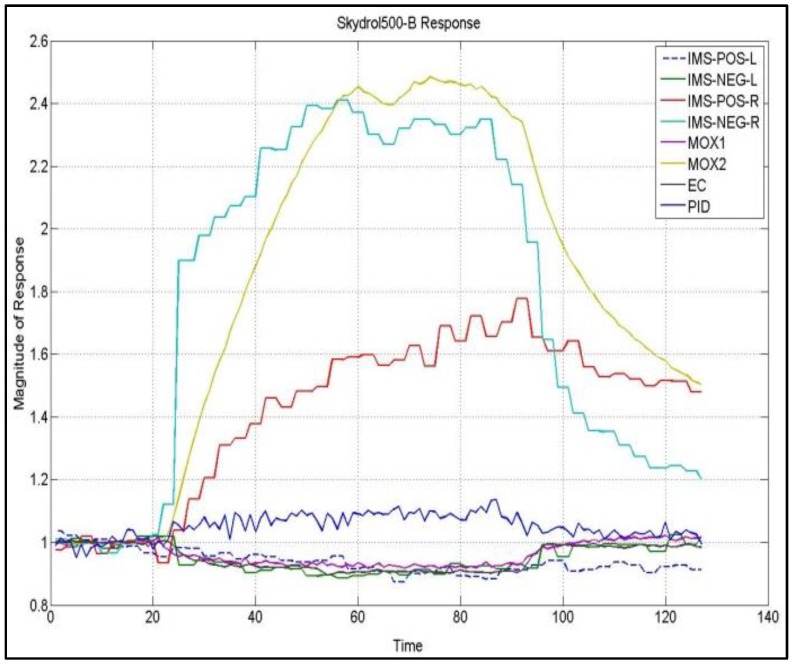
E-nose virtual sensors normalized response upon exposure to a contaminated Composite Fiber Reinforced Polymers (CFRP) panel.

First approach with this paticular machine olfactive scenario began with a commercial electronic nose platform. The Airsense Gas Detection Array (GDA2) was selected for the purpose. The GDA2 is a continuous sampling e-nose designed to operate in harsh environment for the quantification of dangerous chemical compounds. It features a hybrid sensor array, including two Metal Oxide (MOX) sensors, one Electrochemical (EC) sensor, one Photo-Ionization Detector (PID) sensor and an integrated Ion Mobility Spectrometer (IMS) sensor. This array composition thus joins the volatile quantification capabilities of the PID to the discriminative power of the IMS sensor complemented by the broad sensitivities of MOX sensors. As such, the sensing platform provides eight virtual sensing instantaneous responses, including the four solid-state sensor responses and the four areas under the curve computations of the left and right sections (with respect to the water response peak) of the positive and negative spectra of the IMS. Several adaptations have been performed to customize the overall system to the specific task, the most significant affecting the sampling architecture and the measurement methodology. An IR emitter has been used to slightly increase the temperature of the CFRP sample, in order to speed up volatiles desorption resulting in increased uptake from the e-nose; at the same time, the measurement methodology has been modified to resemble the sampling procedure of a typical lab based e-nose, including a baseline acquisition phase, a sensor exposure phase and a flushing/desorption phase. In Figure 1, a typical response to a sample of a contaminated panel of the eight virtual sensors array has been reported.

### 2.2. The SNIFFI e-Nose

SNIFFI is a prototype of a multi-sensor system based on a hybrid array of chemiresistors. It is designed by ENEA to work, not only in a controlled atmosphere, but also as a portable system based on artificial olfaction techniques for gas analysis in real scenarios, for medical, aerospace, food industries and research purposes.

This system returns a graphic visualization of the analyzed sample, created from the matrix formed by the differences between the sensors outputs. By adopting both heated and unheated chemiresistors, as well as different materials and surface functionalizations, the differences of the sensor kinetics (Patterns) can be correlated to the analyzed scent. SNIFFI firmware is equipped with automatic smelling recipes, which provide repeatable measurements and optimize the sensor responses to the air sample. The design allows analyzing air sample either enclosed in bags or coming from flat and rigid surfaces. In the last case, an IR emitter can slightly heat the surface to improve the desorption of the Volatile Organic Compounds (VOCs) to be detected. The core of the system is represented by the Sensor Chamber that is equipped with a mix of commercial sensors and unheated chemiresistors based on nanostructured semiconductors paste. SNIFFI is compact, stand-alone, lightweight and rugged laboratory equipment whose hardware modularity is designed for upgrades involving innovative technologies and/or new requirements.

#### 2.2.1. System Layers Architecture

**Figure 2:**
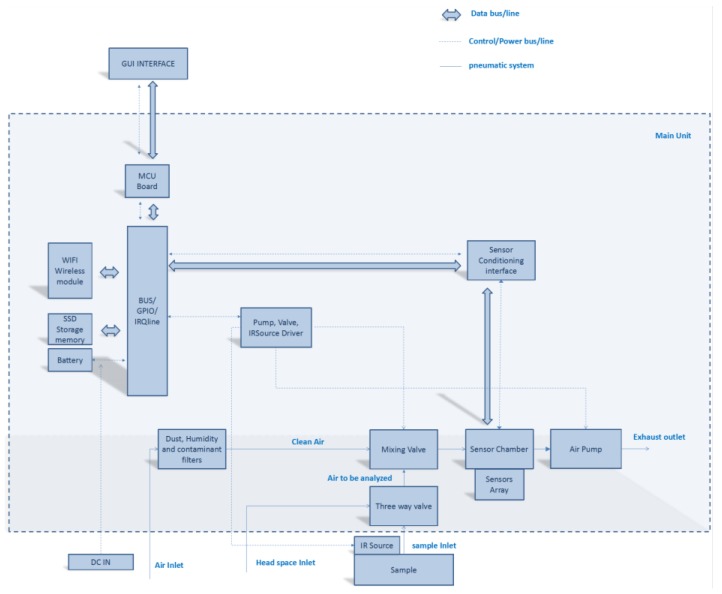
Functional block diagram of SNIFFI system.

**Figure 3:**
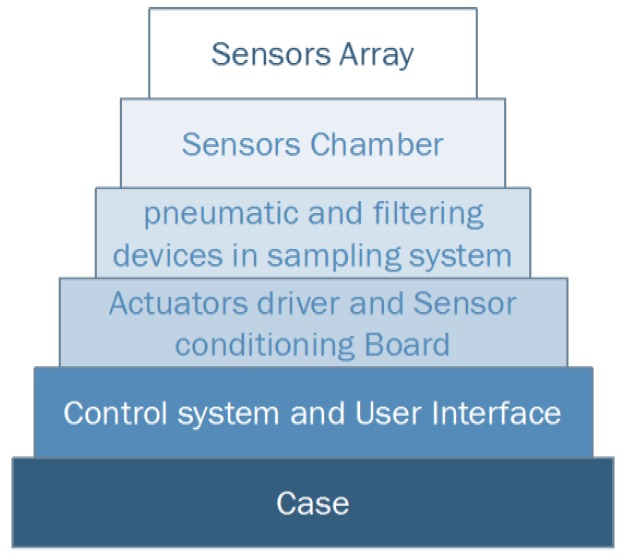
SNIFFI system Layers Architecture.

Being SNIFFI is a closed gas sensing system, the sensor array is confined in a chamber wherein the air sample enters through an air pump. For the sake of experiment repeatability, it is necessary to maintain the array sensor, when not exposed, in an unruffled state by flushing clean air, so that deviations from an equilibrium state can be recorded and correlated to the gas sample analysis. In Figure 2 are shown the SNIFFI main components along with their functional connections, its embedded architecture being organized in different layers, each one requiring different expertise as shown in Figure 3:

- Sensors Array
- Sensors Chamber
- Actuators driver and Sensor conditioning Board
- Pneumatic and filtering devices in sampling system
- Control system and User Interface
- Case

#### 2.2.2. Sensors Array

The sensor system is compatible and can be equipped with six heated chemiresistive commercial sensors (Figaro, MICS, etc.) plugged into four-pin receptacles. Currently, three couples of Figaro Sensors families (Tgs2600, Tgs2602, and Tgs2620) are used. These metal oxide (MOX) sensors are non-specific and suitable for air quality estimation since they exhibit high sensitivity to several chemical compounds at ppb to ppm level in controlled atmosphere.

**Figure 4:**
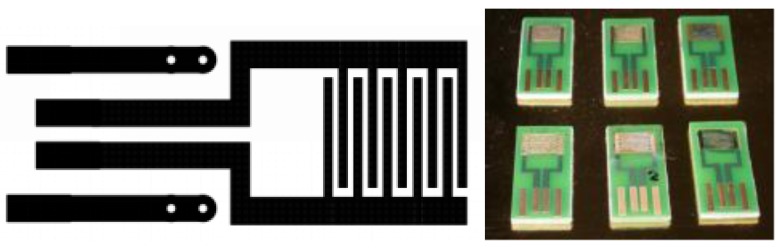
Interdigitated transducer pluggable on USB-like socket.

Temperature and humidity and the total VOCs content are measured by a PID-AH (Alphasense Inc., Great Notley, UK) sensor [20], which completes the commercial section of sensor array. On this system it is also possible to install six chemiresistive prototype sensing films operating at RT. To facilitate the installation of these devices on the Sensor Board, plug & play transducers were specially designed. The connection design (Figure 4) allows inserting the device into a universal USB-like connector in sensor chamber.

The six FIGARO MOX sensors are in a metallic standard TO-5 package. Table 1 illustrates the FIGARO sensors that were qualified for the use in the SNIFFI sensor array.

The unheated devices (see Table 2) are based on various sensing materials synthesized in our laboratory as below reported:

- Graphene LPE (GR_VN)
- Palladium decorated graphene (2 device GR2409)
- Nanostructured Polyaniline (PANI V2d_2ott)
- Polyaniline doped with p-toluene sulfonic acid (PANI-TSA)

Polyaniline solutions were prepared according to a method described in Ref. [21]. In brief, polyaniline emeraldine salt was diluted in tetrahydrofurane (1.53 wt %) for the fabrication of PANI sensors. The PANI doped with p-toluene sulfonic acid (TSA) was prepared by mixing polyaniline emeraldine base and p-toluene sulfonic acid (TSA) in chloroform. Graphene and decorated graphene were synthetized through a simple and eco-friendly chemical process [22,23,24]. All the sensing devices were fabricated by drop-casting few microliters of the suspensions onto transducers and dried in air.

#### 2.2.3. Sensors Chamber and Sampling System Design

**Figure 5:**
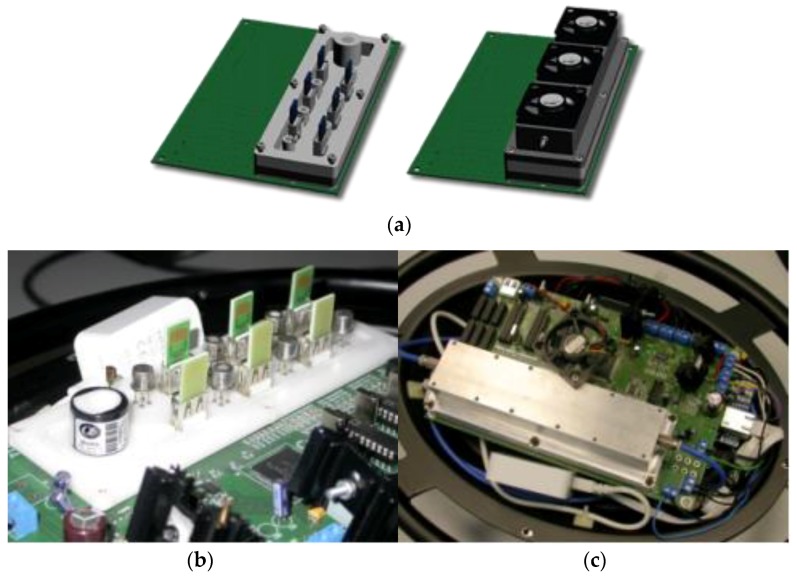
Sensor chamber installed on the sensor board: rendering and photo (**a**,**b**) of the open SC with the 12 chemiresistor disposed in two rows plus the PID (cylinder on the top) and T, RH sensors; and (**c**) an image of the closed chamber.

The rectangular sensor chamber is realized in aluminum with the base in Teflon (Figure 5). Teflon is a good electrical insulator for the sensor board and aluminum ensures a good heat-dissipation.

The air volume is minimized in order to reduce sensor response inertia and 15 sensors are designed to be placed in two rows with six chemiresistors, a Photo Ionization Detector (PID), and temperature and humidity sensors. To prevent damages in custom sensors, the chamber is arranged for mounting aluminum heat sinks and/or fans to enhance the heat dissipation.

**Figure 6:**
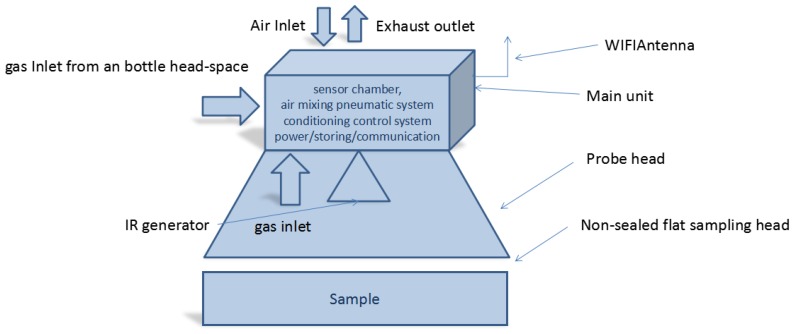
Block design of SNIFFI system.

**Figure 7:**
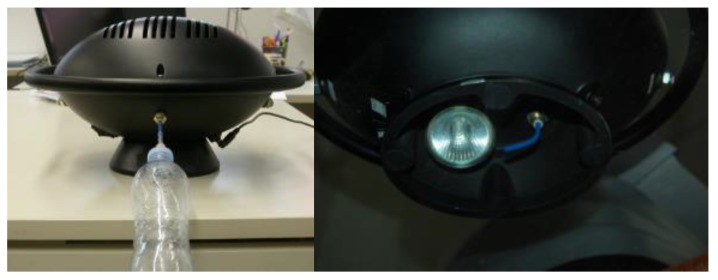
SNIFFI air inlets: (**left**) a front inlet head-space such as a bottle or sampler bag; and (**right**) a bottom inlet provided by a halogen lamp to analyze surface contamination.

As shown in functional block diagram in Figure 2 and block design in Figure 6, SNIFFI has two selectable inlets to perform different sampling applications:

- A front inlet for air sampling of odors coming from an head-space such as a bottle or coming from an air sampler bag (Figure 7);
- A bottom heated inlet to analyze surface contamination, equipped with an halogen lamp for the surface heating to improve the chemical desorption (Figure 7); and
- A purge line with a cleaned airline to perform cleaning phases, maintain gas sensors in a stable steady state and dilute air sampling to avoid sensors signal saturation or contamination.

Main pneumatic devices are:

- Continue current air pump
- Proportional valve (for mixing action)
- Three way valve for inlet selection
- Filtering system cartridge based

#### 2.2.4. Casing, Control System and User Interface

The Actuators and Sensors conditioning Board is driven by an I2C serial bus communication high level logic control layer based on an Arduino Microcontroller. This low cost and time saving Open Source Hardware solution allowed to deploy a Graphic User Interface, based on a web server for manual machine control, also permitting to call and save smelling recipes, the WiFi access and transmission of sensors log. Android and Java packages are available for multiplatform systems (tablet and smartphone). In the future, this configuration can be easily exported to new generation Open Source Hardware Single Board Computers with a small form factor (Raspberry, Arduino, etc.).

**Figure 8:**
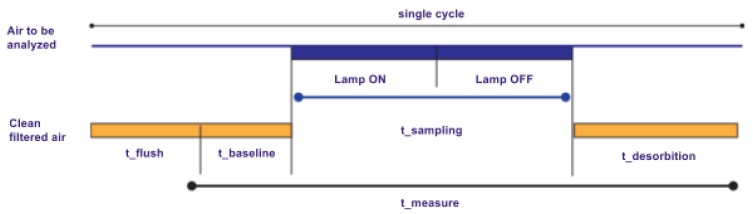
The SNIFFI generalized measurement cycle.

Repeatability of sampling analysis is guaranteed by automated smelling recipes or measurement cycle. The measurement cycle can be repeated N times and it is divided into four phases, the duration of which can be set by the user by means of the graphical interface (Figure 8).

**Figure 9:**
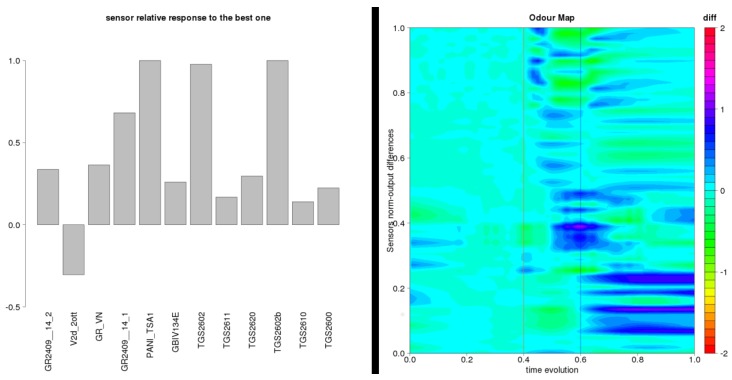
SNIFFI output Graphical representation to exposure of a contaminated panel: (**left**) histogram of the normalized maximum response of each sensor; and (**right**) the odor pattern of the sample analyzed.

Log files, stored in internal solid state memory, can be rapidly visualized with an R script that can graph intensity and smelling pattern, as shown in Figure 9.

**Figure 10:**
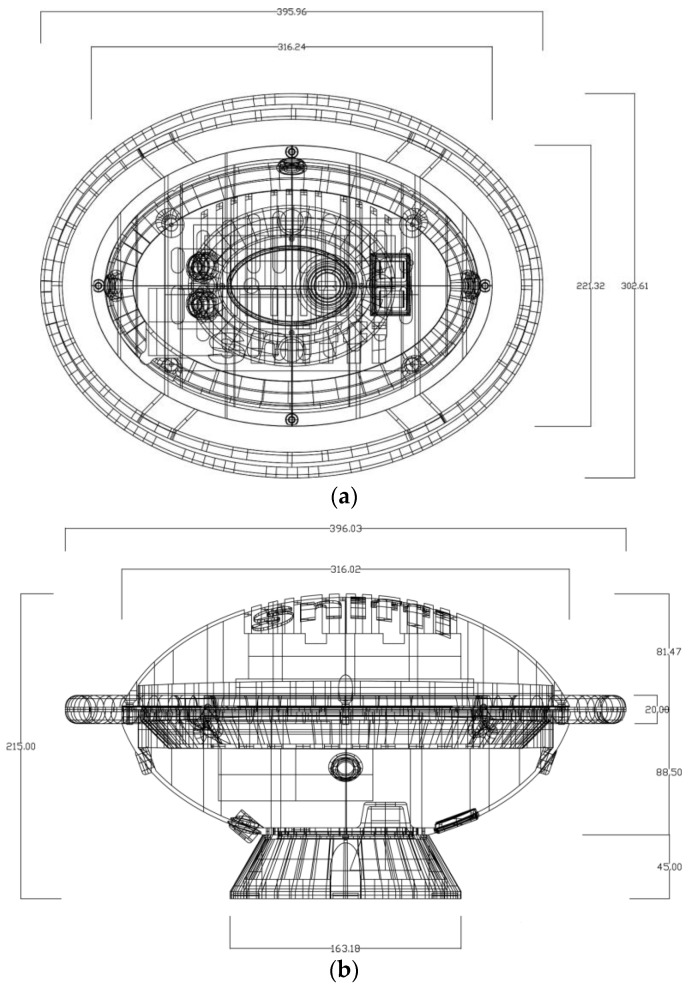
SNIFFI appearance and dimension (in mm, top (**a**) and side (**b**) view of device).

The designed housing (www.monicamassera.com) is made of aluminum to assure the best heat dissipation. The design combines the hardware disposition with an easy access to the sensor chamber. The ovoidal shape maximizes the mechanical strength and the ergonomic shape of supports to facilitate its mobility (Figure 10).

## 3. Experimental Methods

### 3.1. Contamination Scenarios

Several CFRP contaminations and damage may occur during aircraft assembly and maintenance operations. In this framework, we refer to a list comprising hydraulic fluids/water, release and deicer agents and moisture contaminants completed by thermal damages. The list was actually defined by partners of EU ENCOMB project that including AIRBUS consortium, the main EU Aerospace industry stakeholder. Specifically, the hydraulic fluid herein considered is a fire-resistant phosphate ester liquid, commercially known as Skydrol^®^500-B. In particular conditions, it can release phosphoric acid and alcohols that actually undermine the CFRP structure; release agents are silicon based products used for the molding process of composite panels. However, contamination from this silicone material can penetrate up to hundreds of nm into the CFRP panel matrix, severely affecting any further adhesive bonding of the panel itself. Contamination from hydraulic fluids, moisture uptake and also release agents can lead to the presence of volatile compounds or even to a change of their composition within the CFRP surfaces. Solvent residues or even reaction products (as in the case of Skydrol^®^500-B) can indeed be left onto the CFRP surfaces and can be detected by a gas-phase analyzer. Likewise, moisture contamination can be detected by a local variation of the humidity level of the sample during IR stimulated heating.

The CFRP material that has been adopted throughout all the conducted studies is a thermoset matrix with carbon fibers arranged in unidirectional layers (HexPly© M21 matrix from Hexcel and T700 low density carbon fibers).

In a first assessment of the e-nose suitability for these contamination scenarios, conducted during the ENCOMB project, the CFRP samples were subjected to significant contamination levels, on the highest ranging edge of what can be expected to be found in real assembly or maintenance scenarios. This can hence be considered a low TRL setup. The only exception was represented by the moisture contamination scenario in which CFRP samples were exposed to 30%, 75%, 95% and 100% relative humidity (RH %) at 70 °C which reflected normal operating conditions.

The contamination by hydraulic fluid/water mixture was carried out by mixing the hydraulic oil with purified water in volume ratio 1:1 at 70 °C for 14 days. The aqueous phase collected thereafter resulted to be acid (pH 2). This aqueous extract of Skydrol^®^500-B-4 was diluted in order to obtain solutions at pH 3 and pH 4. These three solutions were then employed to contaminate CFRP samples. In detail, four 10 × 10 cm^2^ CFRP specimens were dipped into the solutions: one for each acid solutions and one for a blank solution (pure deionized water) and left in contact with the solution for 672 h at 70 °C.

The contamination with release agents was performed by dip-coating 10 × 10 cm^2^ CFRP specimens into hexane solutions of a commercial release agent (Frekote 700NC) at different loadings from 1% up to 20% by mass. After this process, the silicon concentration on the CFRP surface has been measured by X-Ray Photoelectron Spectroscopy defining the four classes of contamination obtained, 2.1%–2.7%, 6.5%–6.8%, 8.2%–9.5%, and 10.1%–10.4%, as atomic Si percentage.

The moisture contamination was achieved by exposing the CFRP panels to deionized water and to saturated aqueous solutions of K_2_SO_4_, NaCl and MgCl_2_ for 672 h at 70 °C. The water uptakes were then evaluated by the mass increase of CFRP samples with respect to the dry ones. The recorded relative mass uptake was 0.29%, 0.79%, 1.08% and 1.26% for exposure to RH 30%, 75%, 95% and 100%, respectively.

Currently, the assessment protocol used during ENCOMB project has been mainly chosen on the basis of simulation studies and then carried out in laboratory in very controlled settings. CFRP samples have been subjected to significant contamination levels in the controlled environment. These conditions were far from what can be expected to be found in real operative scenario. As such, the TRL of these techniques was found to stay in the low (1–3) end of its scale.

The new COMBONDT project is aimed to uplift and extend the technology readiness level (TRL) of selected technologies for pre- and post-bond cleaning state inspection of CFRP panels in the primary aircraft structures. In order to raise it, the contaminated test samples should be adapted in a way to be much more similar to real operating condition contamination scenarios. The main relevant scenarios for CFRP panels bonding quality assurance are aircraft *production*, when panel are firstly bonded during aircraft construction, and *maintenance*, when panels are repaired after operative duties. For each scenario, CFRP panels have been contaminated with different chemicals at different concentration levels. As a general approach, the contamination levels were reduced in order to obtain a loss of bond strength of 30% of the initial strength (of a reference specimen, marked with RE label) for the highest contamination level. In particular, the hydraulic oil spiking was achieved by applying artificial fingerprints onto the CFRP panels with fingers previously dipped in Skydrol^®^500-B. Release agent contamination was performed with the same methodology described above for the low TRL scenario but with lower contaminant loadings. The moisture scenario was basically unchanged unless the limitation to three exposure levels, namely RH at 30%, 75% and 100%.

In the following, we will refer to ENCOMB project scenarios as “Low TRL” scenarios while “High TRL” scenarios will indicate the COMBONDT project ones.

### 3.2. Sample Analysis

In the low TRL scenario, sample analysis was performed by positioning the CFRP samples close to the e-nose gas inlet (actually, not more than 4 mm distance). The extraction of the volatiles from the sample surface was then aided by switching on the IR emitter. The lighting time was different depending on the e-nose employed.

In the case of the high TRL scenario, the low contaminant concentrations have required a further adaptation of the system setup and, in particular, of the sampling system. The one tested herein foresees the use of a low-boiling solvent with the aim to enhance the extraction of volatiles from the surfaces and to differentiate the samples based on their capability to retain and desorb the solvent as a function of the surface contamination. The sampling system method hereinafter indicated as “PC-method” (where PC stands for “Probe. Chemical”), is performed using a chemical probe, i.e., spraying few milliliters of ethanol with an airbrush over the surface of the sample. The subsequent e-nose analysis was then performed within a defined time span (<2 min).

For the sake of repeatability, the environment where the measurements take place also needs to be controlled. For this reason, we designed a closed space in which air conditioning is also equipped with activated carbon filtering set so to cut down unwanted VOCs.

E-nose equipment needs to pass a validation test before starting the sample analysis. The validation test checks that both environmental and sensors parameters fall within fixed windows.

Each CFRP sample is analyzed within two minutes from the Chemical Probe contamination; the analysis is performed at first by SNIFFI, whose results are reported in the next section, and then after by the GDA2. Sampling sequence is random and every hour a test performed on a reference sample ensures that no equipment poisoning is ongoing and the environmental setting is not changed.

### 3.3. Problem Setting

An NDT tool for pre-bond quality checks must ensure the cleanliness state of two panels which should be adhesively bonded. Contamination, if any, should be detected and identified. From the artificial olfaction point of view, the problem may be posed in terms of a multiclass classification with each possible contaminant representing a separated class, also including a class for uncontaminated samples. The decision that the operator has to take is posed in the classical GO-NOGO fashion of NDT quality assurance tools. Eventually, once the contaminant has been identified, it could be quantified by a dedicated subsystem. In the following, we will describe the contamination detection and identification problem.

## 4. Pattern Analysis and Results

### 4.1. Low TRL Settings

During the ENCOMB project, our focus was to detect, identify and quantify the contamination of the base CFRP panel, using the GDA2 electronic nose. Although the contamination levels were significant and capable to elicit a distinct response from the electronic nose, in most of the contamination scenarios the technique was challenged by the request to obtain a fast and dependable response.

These requirements led to the design of an ad-hoc pattern analysis system, featuring the required capabilities (see [19] for further details).

**Figure 11:**
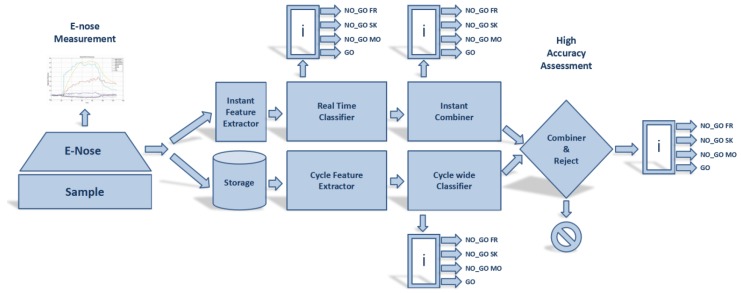
The proposed pattern recognition architecture. A combination law combines the results obtained by two different classification subsystems on the entire measurement cycle.

Our main goal was to extract all the possible information embedded in the response of the sensor array throughout the entire measurement process. At the same time, we needed to achieve a first response as soon as possible while retaining a high accuracy. The possibility to express the confidence with which the classification decision was taken was mandatory for dependability requirement. Eventually, samples with low classification confidence could be rejected and prompted for further analysis with a different technique. Figure 11 depicts the detailed architecture including two main subsystems. The data flow is, in fact, split in two main streams. The first one, a Real Time classifier (RTC), is focused on the analysis of instantaneous responses of the sensors array by a real time classifier disregarding temporal information. A partial combination system will take care of fusing outcomes of instantaneous classification outputting a continuous, on-line, combined classification result based on all the previous instantaneous decisions. A second subsystem, named as Dynamic Classifier (DC) is instead devoted to the analysis of the entire cycle response of the e-nose to the measurement process. This typical subsystem will base its decision on the values of features that depend on specific parts of the measurement process, e.g., the steady state response or the first derivative of the sensor signal during abrupt exposure to the relevant volatiles. Waiting until the end of the measurement process, the operator can benefit from the output of a combiner that will take care of combining the decisions coming from the two different subsystems. The underlying hypothesis is that these two at least partially independent and complete problem “views” may be combined with positive outcomes. By analyzing the confidence with which the two subsystems have produced their final decision, it is possible for the final combination stage to refuse to classify a sample because of the low confidence with which this decision could be made. This will allow the operator to choose to resort to further analysis before qualifying or not the panel under analysis.

The final classification decision has been achieved taking into account classification confidence rating (CR) associated to each class by both subsystems with a *mean* rule:

(1) $$Comb_{CBCCycle}\left(x_{i}\right)=\frac{CR_{RTCcycle\;}\left(x_{i}\right)+CR_{DCcycle}\left(x_{i}\right)}{2}\;i\in \left[1,\;2,\;3,\;4\right]$$

where *x* is the panel under classification, *i* is the contamination class, [1, 2, 3, 4] is the set of labels for each contamination class, and *CR_RTCycle_* and *CR_DCCycle_* are CR levels associated to a cycle wide decision expressed by, respectively, the Real time and Dynamic classifier:

(2) $$CR_{RTCcycle}\left(x_{i}\right)=\frac{\sum_{j=1}^{T}ON_{ij}}{\sum_{j=1}^{T}\left(\sum_{i=1}^{4}{\mathrm{ON}}_{ij}\right)}\;i\in \left[1,\;2,\;3,\;4\right]$$

(3) $$CR_{DCcycle}\left(x_{i}\right)=\frac{ON_{i}}{\sum_{i=1}^{4}ON_{i}}\;i\in \left[1,\;2,\;3,\;4\right]\;$$

in turn, $ON_{i}$ is the Output Neuron vote associated to *i*-th contamination class, $ON_{ij}$ is the Output Neuron vote associated to *i*-th contamination class at the time *j*, and *T* is the total number of samples in a measurement cycle.

Finally, the combined classification decision, i.e., winner contamination class $WC_{CBcycle}$, associated to the panel *x* is identified by means of a majority voting scheme as the class maximizing the equations:

(4) $$WC_{CBcycle}\left(x\right)=\underset{i\in \;\left[1,\;2,\;3,\;4\right]}{\max}\left(Comb_{CBCcycle}\left(x_{i}\right)\right)$$

Intermediate cycle classification results, $WC_{RTCcycle}$ and $WC_{DCcycle}$, are computed as the class maximizing the amount of instantaneous classification votes,

(5) $$WC_{RTCcycle}=\underset{i\in \left[1,\;2,\;3,\;4\right]}{\max}\left(\overset{T}{\underset{j=1}{\sum }}ON_{ij}\right)$$

and

(6) $$WC_{DCcycle}\left(x\right)=\underset{i\in \;\left[1,\;2,\;3,\;4\right]}{\max}\left(ON_{i}\left(x\right)\right)$$

i.e., the most active *ON* class.

**Figure 12:**
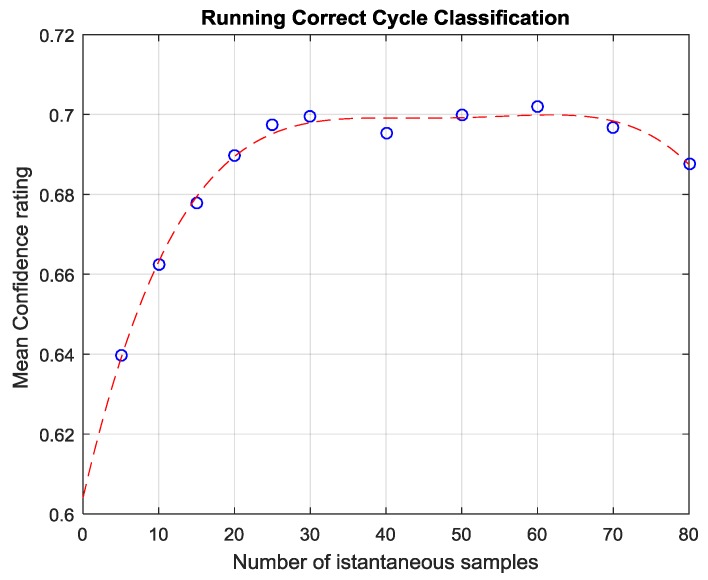
Confidence Rating associated to winning class expressed by Real Time classifier (Equation (2) during the length of a measurement (averaged across all correctly classified CFRP samples). The confidence rating consistently increases as new instantaneous olfactive patterns are evaluated and finally reaches a pseudo-steady state after 25 s.

**Figure 13:**
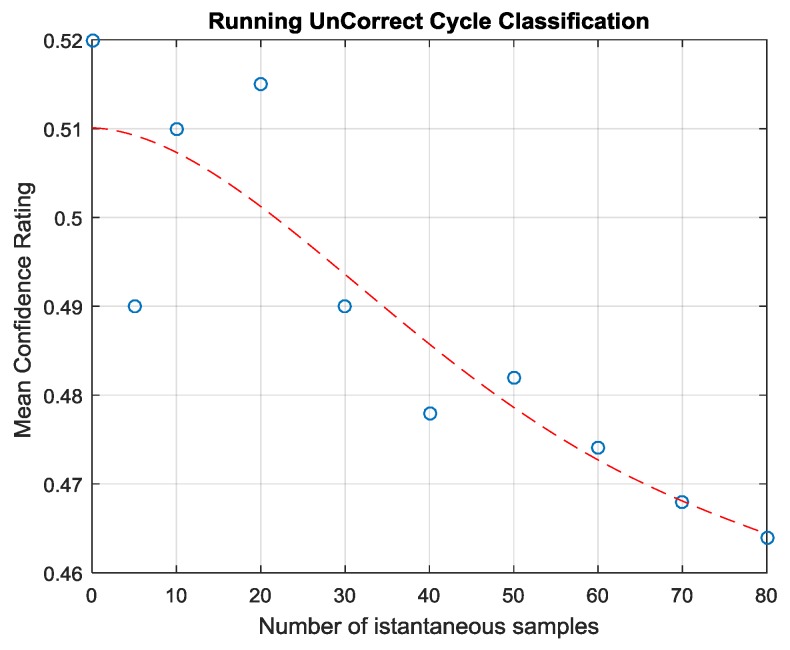
Confidence Rating expressed by Real Time classifier (Equation (2)) averaged on all incorrectly classified samples during the length of a measurement cycle. The confidence rapidly falls as the number of evaluated instantaneous olfactive patterns increases from 20 s onward.

Note that Equation (2) can be computed in an on-line, running fashion, while the measurement process takes place. In fact, the maximum confidence value among the classes appears to stabilize to higher values after just 20 s when the correct decision is taken; conversely, when misclassification occurs, the confidence signal will decrease over time (see Figure 12 and Figure 13). In this way, Equation (2) can provide a very fast estimation of the contamination status of the panel (for more details see [19]).

Applying this system to the low TRL dataset depicted in the previous section has led to the confusion matrix depicted in Table 3.

**Figure 14:**
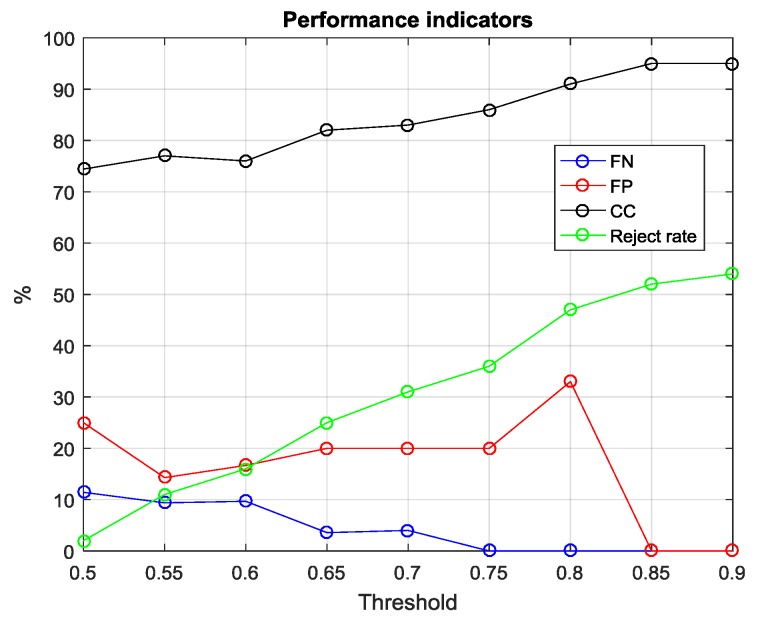
False Negative rate (blue), False Positive rate (red), and Correct Classification rate (black) as a function of reject threshold values. When threshold value increases, FP and FN rate go to 0, while Reject rate and CC increase significantly. Currently, in order to keep reject rate under the 20% range, we must accept an up to 5% FN rate.

On the hypothesis that the overall confidence associated with incorrect classification is lower on average than the one associated with correct classification acts, we set a threshold on confidence ratings in order to reject those samples that elicited a low classification confidence. Figure 14, finally, depicts the relationship between percentage performance indicators in function of the threshold values.

### 4.2. High TRL Settings

As previously stated, two operative settings have been chosen for tests in COMBONDT project: maintenance and production. The measurement protocol has involved the use of both the previously described e-nose platforms: the Airsense GDA-FR Electronic Nose and the ENEA e-nose Prototype, namely SNIFFI. The two different sampling methods (“0-Method” and “PC-Method”) have been implemented in order to detect contaminants preventing any negative impact on the CFRP sample.

In total, 174 measurements have been collected (see Table 4) in order to build adequate datasets for the different scenarios and sampling methodologies.

As we can see, the number of the implemented measurement cycles is significant, although still limited for an electronic nose based typical tuning process. The number of possible measurement repetitions is also severely affected by time (the delay with which we perform the measurement) and by the use of the PC method that may effectively remove a significant part of the contamination residual hence reducing the capability of the electronic nose to grasp the variability in the sensors response.

Whatever adopted method, contamination scenario or e-nose, sampled measurement data have been processed to extract consistent information able to describe the variability of e-nose response with a limited set of values. For the SNIFFI e-nose, we only take into account MOX and PID sensors. Table 5 shows the feature extracted from all the relevant signals.

Selected features try to highlight both the dynamic and steady state behavior of each sensor’s response in the SNIFFI e-nose array as prompted by relevant literature ([25,26]).

In this way, for each scenario, we have obtained a significant number (Table 6) of descriptive features summarizing the SNIFFI response to contaminated panel.

On the other hand, from each of the eight GDA-FR e-nose signal responses, the extracted features have been reported in Table 7, while Table 8 summarizes the dimension of the related dataset.

In order to check if the above extracted features actually capture the variability of the e-nose response and the differences in the type and level of samples contamination, a principal component analysis (PCA) has been performed on the each above datasets normalized per column (sensor) to zero mean and unitary variance.

Since the results of the production scenario were not satisfactory, in the next sections we will focus on the maintenance scenario reporting the scatter plots of the PCA scores in the first two (or three) components plane in order to compare discrimination capability of each e-nose when working with the two different sampling methods. The scores relating to each contaminant and contamination level were clustered. Centers of the clusters (mean scores) grouping single contamination points are highlighted as well as one-sigma ellipsoids helping to define the significance of the observed differences in clusters localization. Particularly, the two-dimensional ellipses were drawn using the following equations:

X = mean(A) + std(A) × cos t,

Y = mean(B) + std(B) × sin t

were A and B, respectively, are the scores related to two of principal components, “std” is the standard deviation and t is the parameter in [0, 2π].

Further analysis will include the use of machine learning algorithms focused on the assessment of the capability to detect and discriminate contaminants.

#### Maintenance Scenario

In the maintenance scenario, panels are repaired after operative duties. After the detection of damage, the panel is locally scarfed to remove the outer damaged layers. These are then substituted with a patch that is bonded over the scarfed area. In order to simulate this scenario, CFRP panels have been contaminated by means of artificial fingerprints of Skydrol^®^500-B aviation hydraulic oil (marked as FP), and potassium formiate based de-icer fluids (DI). Otherwise, the panel surface has been exposed to high temperatures, generating thermal degradation (TD) of its structure.

**Figure 15:**
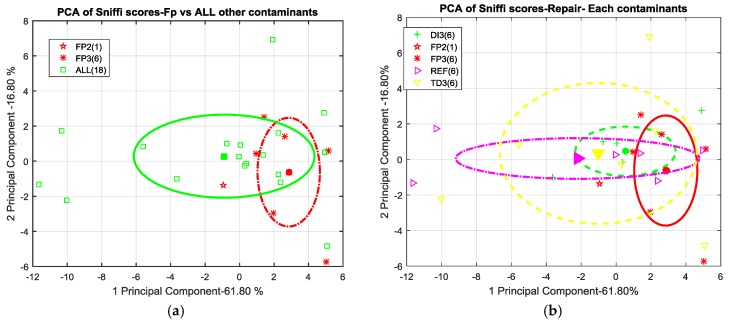
(**a**) 2D scatterplot of PCA scores relating to SNIFFI sensors response in Maintenance scenario sampled by 0-method. Comparison Skydrol^®^ vs. all other contaminant (ALL). (**b**) 2D scatterplot of PCA scores relating to SNIFFI sensors response in Maintenance scenario sampled by 0-method. Each contaminant is depicted with its cluster cloud.

In this scenario, both the e-noses have shown their best results in the detection and discrimination of Skydrol^®^ contamination at the highest concentration level. Essentially, little, if no detection capability has been shown for the other contaminants. Actually, the 2D scatterplot of PCA scores relating to SNIFFI data sampled by 0-method shows weak separability of FP level 3 contamination versus all others contaminants (Figure 15a). Skydrol^®^ cluster cloud appears to be sparsely displaced along these principal components. The significant variance in this space of these samples does not allow for a sufficient one-sigma separability. Likewise, no other contamination seems to come out (Figure 15b). Indeed, the cluster, containing the scores relating to panels subjected to thermal degradation at the highest temperature (TD-3), covers other clusters clouds such as no separability properties are underlined. Probably, this behavior is due to the presence of several outliers in TD-3 contaminated panels.

**Figure 16:**
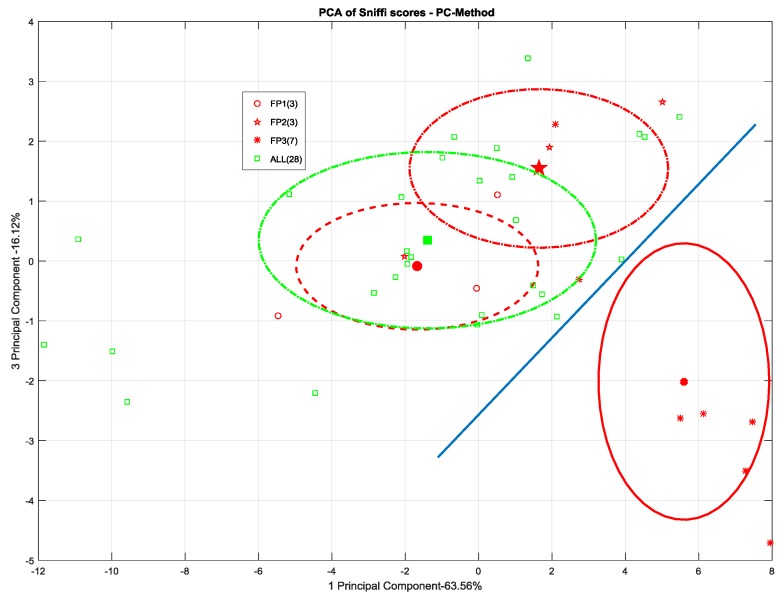
PCA plot (2D) of SNIFFI sensor array response sampled by PC-method.

Conversely, the use of the chemical probe treatment on the surface seems to emphasize its Skydrol^®^ contamination status. As we can see in Figure 16, FP-3 is clearly distinguishable from the cloud of remaining data scores. Moreover, a consistent, more than one-sigma, separation distance appears between FP-3 and the other samples including Skydrol^®^ at the lower concentrations.

**Figure 17:**
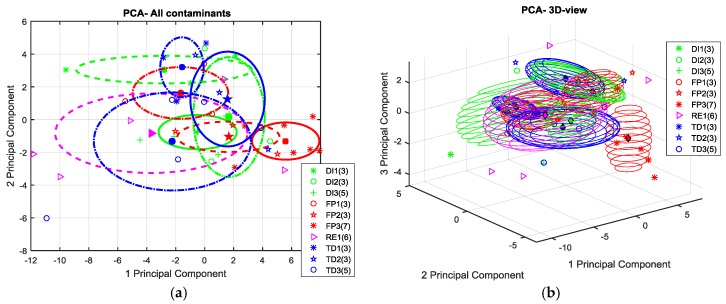
(**a**) 2D; and (**b**) 3D scatterplot of PCA scores relating to SNIFFI response in maintenance scenario sampled by PC-method.

However, as shown in Figure 17, it does not seem possible to consistently detect the other scenarios distinguishing their olfactive patterns from the uncontaminated samples ones. Even in this case, however, at least the FP level 3 cluster pops out from the point cloud representing the otherwise undistinguishable contamination scenarios.

**Figure 18:**
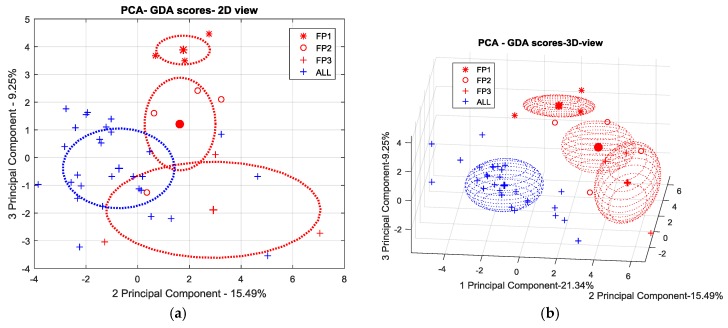
(**a**) 2D; and (**b**) 3D scatterplot of PCA scores relating to GDA sensors response in maintenance scenario sampled by PC-method. Comparison Skydrol^®^ contamination versus all other contaminants (ALL)

On the other hand, the GDA e-nose shows an improved discrimination capability with respect to SNIFFI, being able to distinguish artificial fingerprint contamination samples at each of the three contamination levels (Figure 18b), if PC-method is used.

**Figure 19:**
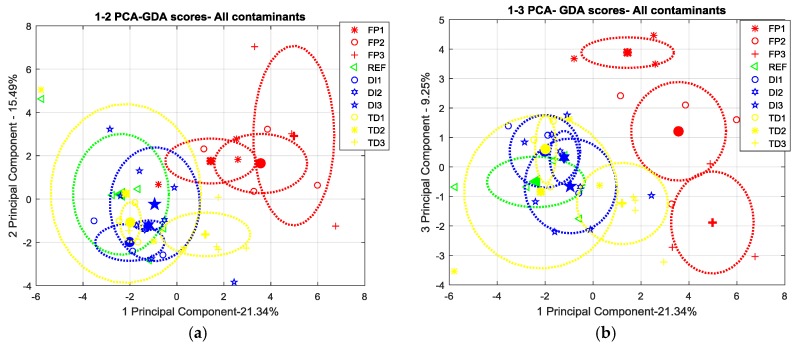
Two different 2Dprojections of PCA scores (**a**): PC1 vs. PC2, (**b**): PC1 versus PC3 relating to GDA sensors responses sampled in maintenance scenario sampled by PC-method. One by one comparison of the contaminants at each concentration level.

In Figure 19, we show that this capability cannot be extended to the other contamination and damage settings.

**Figure 20:**
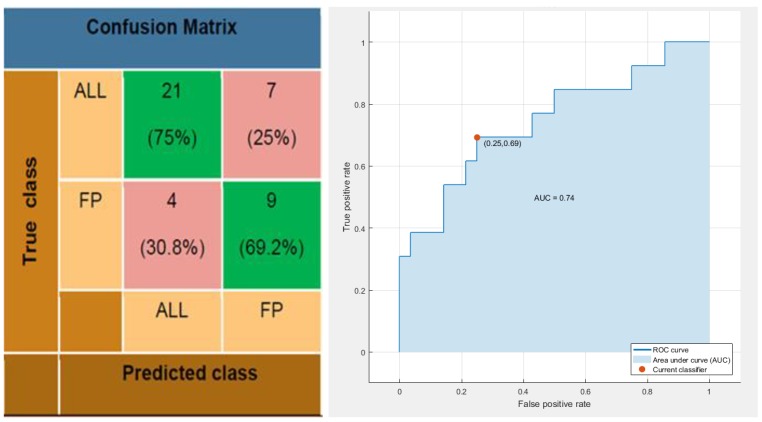
Confusion Matrix and ROC (Receiver Operating Characteristic) Curve of the Linear Discriminant result on SNIFFI response (in maintenance scenario) sampled by PC-Method.

Finally, further analysis by means of classical machine learning algorithms have been focused on the e-nose capability to detect and discriminate FP contaminated panel sampled by PC-method, in maintenance scenario, making use of SNIFFI e-nose. Specifically, a simple linear classifier provides, for the processed SNIFFI response, a correct classification rate of 73.2% with a false negative rate set to 30.8%. Making use of ROC (Receiver Operating Characteristic) curve, we could explore different trade-off between sensitivity and specificity (see Figure 20). Specifically, in the above described setting, the value of area under ROC curve is settled at 0.74 which accounts for a noticeable improvement if we take into account that the system shows limited detection capabilities of Skydrol^®^ at low concentration levels using the first principal components (see Figure 15).

Eventually, our analysis showed that the PC method effectively enhanced the sensibility of both the SNIFFI and GDA2 method towards Skydrol^®^ contamination. Specifically, PC sampling method enhances contaminants clusters separation, mitigating SNIFFI e-nose limitations and allowing detection of Skydrol^®^, at least at the highest contamination level.

## 5. Conclusions

In this paper, we report the results of our research unit, aimed at exploring the possibility to use e-noses as effective NDT tools in a highly demanding scenario such as the modern aerospace industry. This effort, over the years, has led to the design of an ad-hoc platform capable of implementing custom sampling, measurement and data analysis methodologies. Specifically, we have presented a pattern recognition architecture capable of dealing with fast and reliable classification requirements, offering real time assessment of the contamination status, classification accuracy and ensuring a reject option. A new effort is now ongoing to face high TRL requirements in relevant production and maintenance scenarios. Together with the design and results of its novel advanced pattern recognition system, we have shown results of the first part of this new endeavor obtained with samples developed in the H2020-COMBONDT project. A first level data processing architecture has allowed to objectively assess the identification capabilities of the SNIFFI prototype and the customized version of the GDA2 e-noses. While the SNIFFI prototype has proven capable of detecting oily fingerprint contamination only at its highest considered level, the customized GDA2 was proven capable of identifying it even at the lowest levels. These results appear promising for the application of e-nose technology in this high value sector, in particular as a first line tool for hydraulic oil contamination screening before bonding. Further research activities will now focus on the improvement of SNIFFI sensor array diversity and environmental interference rejection as well as on the application of the developed advanced pattern recognition techniques to recorded signals. Simultaneously, we will aim to further scale up the TRL by considering dual contamination scenarios. In this case, CFRP panels will be subjected to contamination by two different agents.

## Figures and Tables

**Table 1:** List of Figaro MOX sensor device that can be mounted on the SNIFFI platform.

Code Name	Sensing Behavior
TGS2602	high sensitivity (sub-ppm) to VOCs and odor Gases and gaseous air contaminants
TGS2611	high sensitivity (sub-ppm) to Methane, Hydrogen and Ethanol
TGS2620	sensitivity to large spectrum of VOC, Carbon Monoxide, Ethanol and Hydrogen
TGS2600	high sensitivity (sub-ppm) to Carbon Monoxide, Hydrogen, Ethanol
TGS2610	high sensitivity (sub-ppm) to LP gas

**Table 2:** List of *unheated* sensor devices, produced in our laboratory, that were actually mounted on SNIFFI.

Code Name	Sensing Behaviour
PANI V2d2ott	high sensitivity (sub-ppm) to NH_3_
PANI TSA	sensitivity to Acetone and H_2_S
GR_VN	high sensitivity (sub-ppm) to NO_2_
GR_Pd 2409	high sensitivity (sub-ppm) to Hydrogen

**Table 3:** Confusion Matrix for the advanced pattern recognition system in the Low TRL scenario.

	Uncontaminated	Release Agent	Skydrol^®^	Moisture
Uncontaminated	7	0	1	1
Release Agent	0	13	0	0
Skydrol^®^	1	0	5	3
Moisture	3	0	2	8

Scoring a correct classification rate of 75%, a false positive rate of 22% and a FN rate of 11%.

**Table 4:** Number of executed measurement for each e-nose and for each scenario and method.

	SNIFFI	GDA
Maintenance-0 Method	25	28
Maintenance-PC Method	41	38
Production-0 Method	25	24
Production-PC Method	44	39

**Table 5:** Description of the extracted features for single sensor of ENEA electronic nose prototype.

	Feature Description
Feature 1 (×7)	Steady State Response (wrt avg baseline)
Feature 2 (×7)	Steady State Response—IROff (wrt avg baseline)
Feature 3 (×7)	Desorption Status (wrt avg baseline)
Feature 4 (×7)	Uptake Derivative
Feature 5 (×7)	Desorption Derivative
Feature 6	Temperature
Feature 7	RH

**Table 6:** Total number of features extracted from SNIFFI measurement data.

	0-Method	Pc-Method
Maintenance	37 × 25	37 × 41
Production	37 × 25	37 × 44

Note: 37 is total number of features: 37 = [5 (features) × 7 (sensors) + 2 (T, RH)] (see Table 5); 25, 41, and 44 are the total numbers of executed measurement for each scenario and sampling method (see Table 4).

**Table 7:** Description of the extracted features for GDA single sensor.

	Features Description
Feature 1	Uptake phase derivative
Feature 2	Steady state response derivative
Feature 3	Desorption phase derivative
Feature 4	Uptake phase average
Feature 5	Steady state phase average

**Table 8:** Total number of features extracted from GDA-fr measurement data.

	0-Method	Pc-Method
Maintenance	40 × 28	40 × 38
Production	40 × 24	40 × 39

Note: 40 is total number of features: 40 = [5 (features) × 8 (sensors)] (see Table 7); 28, 38, 24, and 39 are the total numbers of executed measurement for each scenario and sampling method (see Table 4).
